# SGLT2 inhibitors are associated with reductions in epicardial adipose tissue volume and thickness: a meta-analysis

**DOI:** 10.1038/s41366-026-02110-6

**Published:** 2026-05-27

**Authors:** Shaun Khanna, Elana Kalman, Kaushik Thungathurthi, Udit Thakur, Sheran Vasanthakumar, Sohaib Virk, Aditya Bhat, Andrew Lin, Clare Arnott, Nitesh Nerlekar

**Affiliations:** 1 Cardiovascular Program, The George Institute for Global Health, Barangaroo, NSW Australia; 2 Faculty of Medicine, University of New South Wales, Kensington, NSW Australia; 3 Baker Heart and Diabetes Institute, Melbourne, VIC Australia; 4 Victorian Heart Hospital, Monash University, Clayton, VIC Australia; 5 University of Melbourne, Parkville, VIC Australia; 6 Department of Cardiology, Westmead and Blacktown Hospitals, Westmead, NSW Australia; 7 Department of Cardiology, St Vincent’s Hospital, Darlinghurst, NSW Australia; 8 Department of Cardiology, Royal Prince Alfred Hospital, Sydney, NSW, Australia

**Keywords:** Cardiovascular diseases, Metabolic syndrome

## Abstract

**Background:**

Epicardial adipose tissue (EAT), a metabolically active fat depot surrounding the myocardium, is implicated in the pathogenesis of cardiovascular disease. Sodium-glucose co-transporter-2 inhibitors (SGLT2i) have demonstrated cardioprotective effects beyond glucose lowering, including a modification of EAT. We performed a systematic review and meta-analysis to evaluate the effect of SGLT2i on serial EAT measurement.

**Methods:**

A comprehensive search of PubMed, Medline, EMBASE, Cochrane Library and grey literature (English only, 2000–2025) was performed for studies assessing EAT pre- and post-SGLT2i treatment, or SGLT2i vs conventional therapy, using computed tomography, echocardiography, or cardiac magnetic resonance. Data were meta-analysed using random-effects models with standardised mean differences (SMD) as summary effects.

**Results:**

A total of 11 studies, including 284 patients, were included (89 with echocardiographic EAT thickness assessment and 195 with volumetric measurement using CT or CMR). In analyses comparing SGLT2 inhibitors with conventional antidiabetic therapy, treatment was associated with a significant reduction in EAT (Hedges g − 1.64, 95% CI −2.10 to −1.18; *p* < 0.01). In exploratory within-group analyses, SGLT2 inhibitor therapy was associated with a reduction in EAT from baseline to follow-up (Hedges g − 0.62, 95% CI −0.88 to −0.36; *p* < 0.01). On modality-specific subgroup analysis, reductions were observed in both EAT thickness (echocardiography) (Hedges g − 0.74, 95% CI −1.05 to −0.43; *p* < 0.01) and EAT volume (CT/CMR) (Hedges g − 0.54, 95% CI −0.90 to −0.18; *p* < 0.01), with no significant difference between modalities (*p* = 0.32 for interaction). SGLT2 inhibitor therapy was also associated with modest reductions in body mass index (Hedges g − 0.23, 95% CI −0.43 to −0.03; *p* = 0.03) and systolic blood pressure (Hedges g − 0.32, 95% CI −0.59 to −0.05; *p* = 0.02). Exploratory subgroup analyses suggested larger reductions in EAT in studies evaluating dapagliflozin compared with empagliflozin; however, this indirect comparison was based on a small number of heterogeneous studies and should be considered hypothesis-generating only.

**Conclusions:**

SGLT2i significantly reduces EAT volume and thickness, which may contribute to their broad cardiovascular benefits. Targeting EAT may represent a novel therapeutic approach for mitigating cardio-metabolic risk.

**Study registration:**

PROSPERO CRD42024621242

**Figure:**
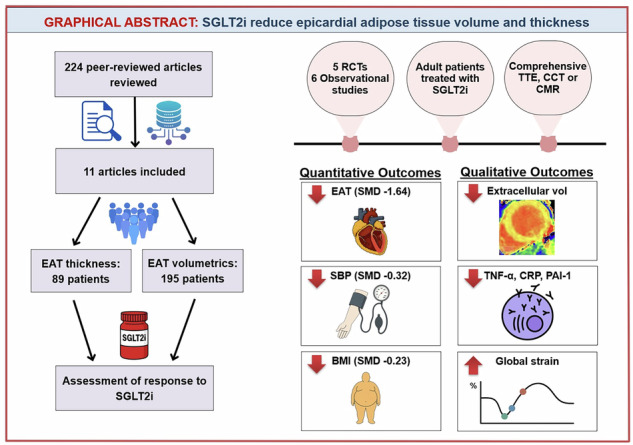
SGLT2 inhibitor therapy is associated with reduced epicardial adipose tissue (EAT) across multimodality imaging (Hedges g −0.62), with consistent effects on both thickness and volume. Modest reductions in body mass index and systolic blood pressure were also observed, alongside improvements in inflammatory and metabolic markers.

## Introduction

Epicardial adipose tissue (EAT) is a metabolically active fat depot located between the myocardium and the visceral pericardium [1]. EAT serves several physiological roles, including mechanical cushioning and energy provision to the myocardium. As EAT comprises up to 20% of total heart mass, it is increasingly recognised as a contributor to cardiovascular pathology when present in excess within a pathological substrate [2]. There is increasing data to suggest that EAT secretes pro-inflammatory cytokines contributing to local myocardial and vascular inflammation [3].

Clinical studies have demonstrated that increased EAT is independently associated with cardiometabolic syndrome and elevated risk of atherosclerotic cardiovascular disease (ASCVD), with correlations with coronary artery disease (CAD) and plaque vulnerability [4–6]. Furthermore, EAT has been linked to atrial cardiopathy, increasing the risk of atrial fibrillation and thromboembolic stroke, and is increasingly implicated in the pathophysiology of heart failure [7]. A positive correlation has also been observed between EAT volume and left ventricular mass and hypertrophy [8].

Sodium-glucose co-transporter 2 (SGLT2i) has emerged as a class of therapies with several cardiovascular benefits, including reductions in heart failure hospitalisation and cardiovascular mortality [9]. The proposed pathways include improvements in cardiac haemodynamics, metabolism, autonomic tone, and inflammation [10]. Notably, SGLT2 inhibitors (SGLT2i) have been shown to reduce EAT volume through attenuation of the associated inflammatory cascade, myocardial fibrosis and adverse ventricular remodelling [11].

Given the growing recognition of EAT as a modifiable risk factor, its quantification has potential utility as a biomarker and therapeutic target. Reductions in EAT have been observed following weight loss, exercise, very-low-calorie diets, and bariatric surgery [12]. Emerging evidence also suggests that treatment with SGLT2i is associated with significant reductions in EAT, though this has only been represented in smaller studies [13]. As such, we conducted a meta-analysis to evaluate the effects of SGLT2 inhibitors on EAT thickness and volume by different imaging modalities, to explore differences in EAT change across different SGLT2i drug classes, and to synthesise additional cardiometabolic effects reported within the included studies. The principal findings and mechanistic implications of this study are summarised in the Graphical Abstract.

## Methodology

### Search strategy and selection

**Fig. 1:**
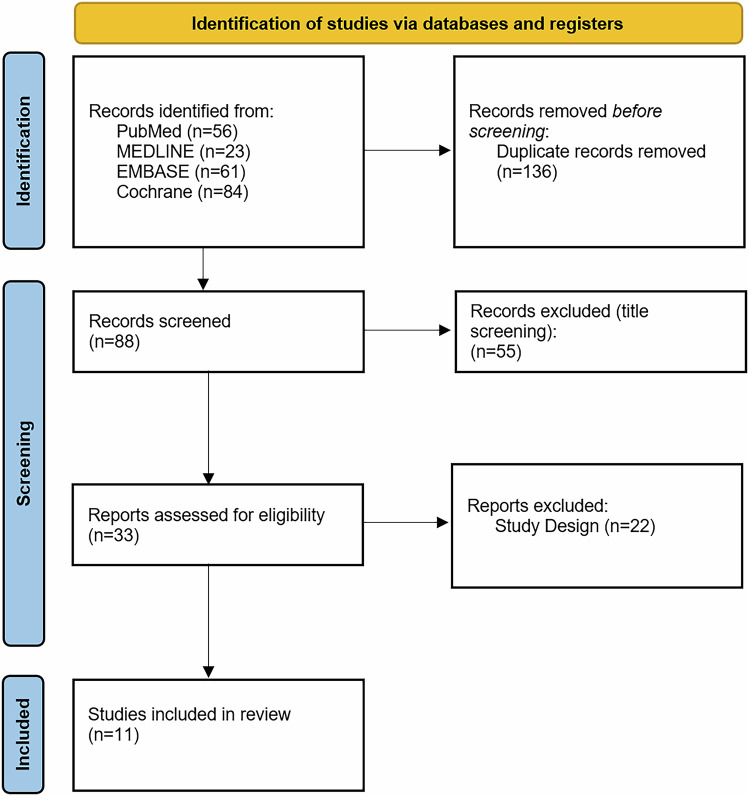
PRISMA flow diagram. PRISMA flow diagram of study selection. A total of 224 records were identified from databases. After removal of duplicates (*n* = 136), 88 records were screened, of which 55 were excluded based on title and abstract. Thirty-three reports were assessed for eligibility, with 22 excluded due to study design. Eleven studies were included in the final analysis.

This systematic review and meta-analysis are reported according to the Meta-analysis Of Observational Studies in Epidemiology and Preferred Reporting Items for Systematic reviews and Meta-Analyses (PRISMA) guidelines and checklist [14, 15]. The review protocol was registered on the International Prospective Register of Systematic Reviews (PROSPERO), registration number CRD42024621242. Figure 1. A systematic literature search of trials of SGLT2i and effects on EAT was conducted using online databases including PubMed, Medline, Cochrane library and EMBASE for studies published from 2000 to 2025. An additional grey literature search identified additional studies that were not identified in the medical databases. Searches were restricted to human studies only. In brief, search terms included: ‘SGLT2 inhibitor OR dapagliflozin OR empagliflozin OR canagliflozin’ and ‘epicardial fat OR epicardial adipose tissue OR subepicardial adipose tissue OR subepicardial fat’ (Supplementary Materials). Grey literature sources included conference abstracts indexed in EMBASE and proceedings available via PubMed, as well as searches of clinical trial registries. Abstract-only publications were screened but excluded if no full-text or quantitative data were available.

Inclusion criteria: (a) adult populations and (b) non-invasive measurements of EAT by cardiac computed tomography (CCT), transthoracic echocardiography (TTE), or cardiac magnetic resonance imaging (CMR) and (c) both pre and post SGLT2i assessments.

Exclusion criteria: Studies were excluded if they were: (a) only in abstract/non-peer review format; (b) did not compare appropriate measures of interest in ≥18 years population; (c) or had missing data.

#### Endpoints

The primary endpoint was change in EAT, with the primary analysis defined a priori as between-group comparisons of SGLT2 inhibitor therapy versus control in randomised and controlled studies. Analyses of within-group pre–post change were performed as secondary (sensitivity) analyses, given their susceptibility to secular trends and regression to the mean. Secondary endpoints included change in blood pressure, body mass index, cardiomyocyte volume, total body subcutaneous and visceral adipose tissue, tumour necrosis factor (TNF)-α, and plasminogen activator inhibitor-1 levels.

### Data extraction

Two investigators (SK and EK) independently screened records retrieved from the search by title and abstract. Details including year of publication, study design, participant characteristics, age, sample size, sample stratification, outcome measures, results and author’s conclusion were collated and examined. Selected records were further screened for eligibility in full text by the same investigators (SK and EK). Data collection was performed independently by two investigators (SK and EK) using the same pre-determined template. Discrepancies at any stage of selection were arbitrated by a third author (KT).

### Quality appraisal of the selected studies for the review

Risk of bias was assessed independently by two reviewers (SK, EK). Randomised controlled trials were evaluated using the Cochrane Risk of Bias 2 (RoB 2) tool [16], assessing bias arising from the randomisation process, deviations from intended interventions, missing outcome data, outcome measurement, and selection of the reported result. Non-randomised studies were assessed using the Newcastle–Ottawa Scale (NOS) [17] and additionally evaluated using ROBINS-I [18] to provide a structured assessment of bias due to confounding, participant selection, intervention classification, deviations from intended interventions, missing data, outcome measurement, and reporting bias. The overall certainty of evidence for key outcomes was assessed using the GRADE framework [19], incorporating study design, risk of bias, inconsistency, indirectness, imprecision, and publication bias. Detailed risk-of-bias assessments and GRADE evidence profiles are provided in the Supplementary Materials.

### Statistical analysis

For individual measures of interest, random effects meta-analysis was performed if at least 3 or more studies reported on EAT values. Articles were required to report on a quantitative estimation, including mean, confidence interval (CI) and standard deviation. 95% confidence intervals (CIs) were calculated for all indices. Degree of heterogeneity within the pooled studies was tested using the *I*^2^ statistics and deemed significant if ≥50% [20]. Potential publication bias was assessed visually from funnel plots. In instances where fewer than three studies were available or reporting was insufficient, a qualitative (narrative) synthesis was provided instead.

Where outcomes were reported using different scales or units (i.e. volume vs. linear thickness), standardised mean differences (SMDs) were computed by dividing the mean difference by the pooled standard deviation, allowing for comparability across studies. For pre–post analyses, change scores and corresponding standard deviations were extracted directly from included studies where available. Where change-score standard deviations were not explicitly reported, these were calculated using standard formulae incorporating an assumed within-person correlation coefficient (*r* = 0.5), consistent with standard practice for paired data. Sensitivity analyses were performed across plausible values of *r* (0.3–0.8) to assess the robustness of the findings. Given the use of heterogeneous measurement scales across studies (mm for thickness, cm³ or mL for volume, and g for mass), SMDs were calculated using Hedges *g* to enable comparability across modalities. A test for subgroup interaction was conducted to compare pooled treatment effects between volumetric and linear EAT measurement methods.

Meta-regression analyses were performed to assess the impact of moderator variables on the treatment effect of SGLT2i on epicardial fat. The following baseline characteristics were individually assessed: mean weight, body mass index, systolic blood pressure, LDL-cholesterol, HbA_1_c and eGFR. Statistical analyses were performed using Cochrane Review Manager v5 and STATA/MP version 18.5 (College Station, TX: StataCorp LLC). A two-tailed *p*-value < 0.05 was considered statistically significant.

## Results

### Search results

**Fig. 2:**
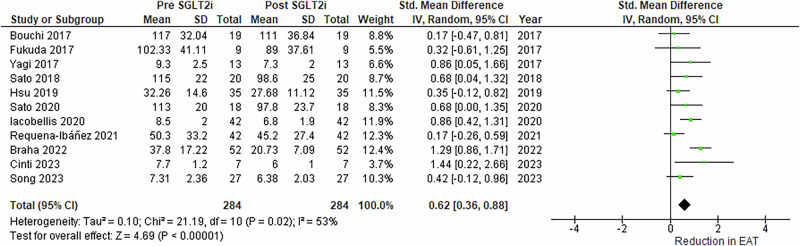
Forest plot (SGLT2i pre and post). Forest plot of within-group change in epicardial adipose tissue following SGLT2 inhibitor therapy. Random-effects meta-analysis demonstrating the effect of SGLT2 inhibitors on epicardial adipose tissue (EAT) from baseline to follow-up across included studies. Overall, SGLT2 inhibitor therapy was associated with a significant reduction in EAT (Hedges g − 0.62, 95% CI −0.88 to −0.36; *p* < 0.01), with moderate heterogeneity (*I*² = 53%).

The initial literature search retrieved 224 articles from various literature sources. After the full screening process, a total of 11 studies met criteria for inclusion in the meta-analysis. Data from these studies with publication dates ranging from 2000 to 2025 were extracted and meta-analysed [21]. A detailed flow diagram with the study selection process and various reasons for exclusion is presented in Fig. 2. Of the 11 included studies [22–31], 6 were observational cohort or single-arm longitudinal studies and 5 were randomised controlled trials. In the RCTs, Sato 2018, Sato 2020, Iacobellis 2020, Requena-Ibáñez 2021 and Cinti 2023, the majority compared dapagliflozin to conventional therapy (80%), and one study compared empagliflozin to placebo. The study characteristics and baseline parameters are summarised in Tables 1 and 2. The certainty of evidence was moderate for randomised data and low for observational studies (Supplementary Materials).

**Table 1:** Study summary.

First Author	Study cohort	Country	SGLT2i/dose	Study design	Baseline EAT	Follow-up EAT	Follow-up period	Imaging method
Yagi^27^	T2DM	Japan	Canagliflozin 100 mg	Observational cohort study	9.3 ± 2.5 mm	7.3 ± 2 mm	6 months	TTE Thickness
Fukuda^29^	T2DM	Japan	Ipragliflozin 50 mg	Observational cohort study	102.33 ± 41.11 cm^3^	88 ± 37.61 cm^3^	3 months	CMR volume
Bouchi^23^	T2DM	Japan	Luseogliflozin 2.5–5 mg	Single arm pilot study	116.33 ± 32.04 cm^3^	111 ± 36.84 cm^3^	3 months	CMR volume
Sato^26^	T2DM	Japan	Dapagliflozin 5 mg	RCT	115 ± 22 cm^3^	108 ± 25 cm^3^	6 months	Cardiac CT volume
Hsu^25^	T2DM	Taiwan	Empagliflozin 12.5–25 mg	Observational cohort study	32.26 ± 14.60 g	27.68 ± 11.12 g	6 months	CMR mass
Sato^21^	T2DM	Japan	Dapagliflozin unspecified	RCT	113 ± 20 cm^3^	110 ± 27 cm^3^	6 months	Cardiac CT volume
Iacobellis^30^	T2DM	United States	Dapagliflozin 10 mg	RCT	8.5 ± 2 mm	8.1 ± 2.4 mm	5.5 months	TTE thickness
Braha^28^	T2DM	Romania	Dapagliflozin 10 mg	Observational cohort study	37.80 ± 17.22 cm^3^	20.73 ± 7.09 cm^3^	6 months	TTE + Cardiac CT volume
Requena-Ibáñez^22^	HFrEF	United States	Empagliflozin unspecified	RCT	50.30 ± 33.20 mL	45.20 ± 27.40 mL	6 months	CMR volume
Song^31^	T2DM	China	Dapagliflozin 10 mg	Observational cohort study	7.31 ± 2.36 mm	6.38 ± 2.03 mm	6 months	TTE thickness
Cinti^24^	T2DM	Italy	Dapagliflozin 10 mg	RCT	7.7 ± 1.2 mm	6.0 + 1.0 mm	1 month	TTE + Cardiac CT thickness

Summary of included studies evaluating the effect of SGLT2 inhibitors on epicardial adipose tissue. Characteristics of included studies, including study design, population, SGLT2 inhibitor type and dose, imaging modality, and baseline and follow-up epicardial adipose tissue (EAT) measurements. EAT was assessed using multimodality imaging, including transthoracic echocardiography (TTE), cardiac computed tomography (CT), and cardiac magnetic resonance (CMR), with measurements reported as thickness, volume, or mass.

**Table 2:** Baseline study characteristics.

First author	SGLT-2	Age	Male gender (%)	HTN (%)	HChol (%)	CAD (%)	Smoking (%)	BMI	SBP mmHg	HBA1c (%)	LDL Mg/dL	eGFR ml/min
Yagi^27^	Treated (*n* = 13)	62 ± 12	38	62	54	31	23	27 ± 5	131 ± 16	7.1 ± 0.5	106 ± 36	65 ± 19
	Control (*n* = 0)											
Fukuda^29^	Treated (*n* = 9)	66 ± 8	67	-	-	-	-	-	135 ± 16	7.2 ± 0.6	113 ± 40	80 ± 17
	Control (*n* = 0)											
Bouchi^23^	Treated (*n* = 19)	55 ± 12	74	-	-	-	-	-	137 ± 14	7.5 ± 0.7	-	70 ± 14
	Control (n = 0)											
Sato^26^	Treated (*n* = 20)	68 ± 4	80	70	-	100	30	26.6 ± 4.6	-	7.2 ± 0.6	91 ± 26	-
	Control (*n* = 20)	66 ± 6	70	60	-	100	25	25.0 ± 3.1	-	7.4 ± 1.1	84 ± 20	
Hsu^25^	Treated (*n* = 35)	63.5 ± 9.7	48.6	82.9	74.3	48.6	11.4	26.6 ± 3.9	132 ± 17	7 ± 1.1	88 ± 22.5	82 ± 19
	Control (*n* = 0)											
Sato^21^	Treated (*n* = 18)	67 ± 5	83	72	-	-	28	26.2 ± 4.8	-	7.1 ± 0.7	-	-
	Control (*n* = 17)	68 ± 7	71	64			29	25.1 ± 3.3		7.3 ± 1.0		
Iacobellis^30^	Treated (*n* = 50)	52 ± 9	42	-	-	-	-	36.6 ± 7.8	127 ± 14	6.8 ± 0.9	104 ± 34	90 ± 23
	Control (*n* = 50)	51 ± 11	40					34.7 ± 6.0	130 ± 11	6.7 ± 0.8	101 ± 35	91 ± 16
Braha^28^	Treated (*n* = 52)	57.5 ± 10.35	61.5	-	-	-	-	34.55 ± 4.79	-	8.7 ± 1.1	-	-
	Control (*n* = 0)	-	-	-	-	-	-	-	-	-	-	-
Requena-Ibáñez^22^	Treated (*n* = 33)	64 ± 12	61	-	-	-	-	29 ± 6	-	5.8 ± 0.3	108 ± 55	80 ± 21
	Control (*n* = 29)	60 ± 14	62	-	-	-	-	30 ± 6	-	5.8 ± 0.5	95 ± 42	83 ± 24
Song^31^	Treated (*n* = 25)	60.08 ± 7.67	44	36	28	-	24	26.29 ± 1.95	131 ± 9	8 ± 0.5	103 ± 26	-
	Control (*n* = 0)											
Cinti^24^	Treated (*n* = 7)	65.1 ± 2.7	100	-	-	40	-	27.8 ± 1.1	145 ± 5.5	7.8 ± 0.2	-	-
	Control (*n* = 7)	67 ± 2.4	57.1	-	-	57	-	29.1 ± 1.2	135.7 ± 5.6	8.0 ± 0.2	-	-

Baseline characteristics of included studies. Baseline demographic and clinical characteristics of participants across included studies, stratified by treatment and control groups where applicable. Variables include age, sex distribution, cardiovascular risk factors, and key cardiometabolic parameters. Missing data are indicated where not reported.

### EAT measurement

The majority of the studies utilised non-contrast cardiac CT or cardiac magnetic resonance imaging (1.5T CMR systems). In general, EAT volume and cardiac function were assessed using a steady-state free precession sequence covering the LV from base to apex. This was manually contoured (epicardial and endocardial LV contours) using dedicated post-processing software (Siemens Medical Solutions). Cardiac CT assessments were ECG gated with only 25% of studies utilising contrast. In general, the superior border for measuring the EAT volume set at the lower surface of the left pulmonary artery origin and inferior border set at the left ventricular apex. EAT volumes were quantified by calculating the total volume of tissue having a CT density (−150 and −30 Hounsfield units). With echocardiography, EAT was identified as the echo-free space between the outer wall of the myocardium and the visceral layer of the pericardium. EAT thickness was measured perpendicularly on the free wall of the RV at the end of systole and averaged after 3 cardiac cycles using the parasternal long-axis view. A detailed summary of EAT measurement is summarised in Table 3.

**Table 3:** Imaging protocol.

First author	Imaging modality	Scanner/system	Measurement method/protocol
Yagi^27^	TTE	High-frequency linear probe (7.5–11 MHz)	Measured at end-systole in the anterior interventricular groove. LAD visualised longitudinally. Thickness = distance from outer myocardial wall to visceral epicardium, measured perpendicular to pericardium. Three cardiac cycles averaged.
Fukuda^29^	CMR	1.5T (Achieva Dual, Philips / Titan, Toshiba) with 32-channel coil	Systole or diastole based on RCA motion. TR 3.2 ms, TE 1.6 ms, flip angle 15°, SENSE 2.5, FOV 330 × 330 × 128 mm, spatial resolution 1.3 × 1.4 × 1.6 mm. Semi-automatic analysis (Ziostation 2) by blinded operator.
Bouchi^23^	CMR	1.5T (Achieva Dual, Philips / Titan, Toshiba) with 32-channel coil	Same parameters as Fukuda. Patient-specific systole/diastole. EFV analysed semi-automatically on Ziostation 2 by blinded operator.
Sato^26^	Cardiac CT	320-slice MDCT (Aquilion One, Toshiba)	CCTA with 0.5 mm slices. –150 to –30 HU. ROI manually traced between pulmonary artery origin and LV apex. Blinded experienced cardiologist.
Hsu^25^	CMR	1.5 T (Trio, Siemens) with 8-channel CV coil	EAT volume from short-axis images at end-diastole. T1 mapping with MOLLI. Cine MRI: ECG-triggered SSFP. MRS: voxel 4×23×20 mm³, TR/TE 550/30 ms, cardiac-gated. Poor-quality images excluded.
Sato^21^	Cardiac CT	320-slice MDCT (Aquilion ONE, Toshiba)	EAT volume from 0.5 mm axial slices, from lower surface of LPA to LV apex. –150 to –30 HU. ROI manually traced. Blinded cardiologist.
Iacobellis^30^	TTE	Not specified	Measured at end-systole on RV free wall (parasternal long-axis view). Echo-free space from myocardium to visceral pericardium. 3 cardiac cycles averaged. Intraobserver reproducibility assessed.
Braha^28^	TTE + Cardiac CT	Philips MX 16-slice CT	High-res axial CT (1 mm, 120 kV, 40 mA). EAT defined as –30 to –190 HU. Analysed with 3D Slicer v4.8.1. EFV and L4VFV calculated and BSA-corrected.
Requena-Ibáñez^22^	CMR	1.5 T (Magneton Avanto FIT, Siemens) with 32-element surface coils	Short-axis cine during end-expiratory breath-holds. EAT contoured on RV/LV in end-diastole using CMR42 v5.6.3. Modified Simpson’s rule. Independent blinded analysis by two cardiologists.
Song^31^	TTE	Vivid E9 (GE Healthcare, USA)	Parasternal long-/short-axis. End-systolic RV free wall thickness. Echo-free space between myocardium and visceral pericardium. Aortic annulus and papillary muscles as landmarks. 3 cardiac cycles averaged.
Cinti^24^	TTE + PET/CT	PET/CT with Siemens scanner (50 mAs, 120 kV, 3 mm slices)	FDG PET/CT analysed with PMOD. SUVmax/mean in pericardial, perirenal, subcutaneous fat. EAT thickness measured on unenhanced CT at thickest RV anterior wall. Blinded dual reader; interobserver variability <5%.

Imaging modalities and epicardial adipose tissue measurement protocols across included studies.

Summary of imaging techniques, scanner systems, and methodological approaches used for epicardial adipose tissue (EAT) quantification across included studies. Variability in imaging modality, acquisition parameters, and measurement protocols is highlighted.

### Meta-analysis summary

#### Primary and secondary endpoints

Of the 11 studies included in the meta-analysis, 7 assessed EAT volume and 4 assessed EAT thickness. Dapagliflozin was the most commonly studied agent (*n* = 6), followed by empagliflozin (*n* = 2), with single studies evaluating canagliflozin, luseogliflozin, and ipragliflozin. A total of 284 patients were included (89 with echocardiographic thickness assessment and 195 with volumetric measurement). All but one study included patients with type 2 diabetes mellitus. Follow-up duration ranged from 1 to 6 months, with 6 months being the most common.

### Primary analysis (Between-group comparisons)

**Fig. 3:**
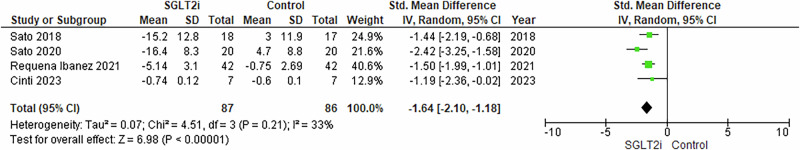
Forest plot (SGLT2i vs controls). Forest plot of between-group differences in epicardial adipose tissue with SGLT2 inhibitor therapy versus control. Random-effects meta-analysis demonstrating the effect of SGLT2 inhibitors compared with conventional therapy on epicardial adipose tissue (EAT). SGLT2 inhibitor therapy was associated with a significantly greater reduction in EAT (Hedges g − 1.64, 95% CI −2.10 to −1.18; *p* < 0.01), with low-to-moderate heterogeneity (*I*² = 33%).

In analyses comparing SGLT2 inhibitors with conventional antidiabetic therapy, treatment was associated with a significantly greater reduction in EAT (Hedges g − 1.64, 95% CI −2.10 to −1.18; *p* < 0.01)(Figs. 2, 3).

### Secondary (sensitivity) analysis: within-group change

In exploratory within-group analyses, SGLT2 inhibitor therapy was associated with a significant reduction in EAT from baseline to follow-up (Hedges g − 0.62, 95% CI −0.88 to −0.36; *p* < 0.01). On modality-specific subgroup analysis, reductions were observed in both EAT thickness (echocardiography) (Hedges g − 0.74, 95% CI −1.05 to −0.43; *p* < 0.01) and EAT volume (CT/CMR) (Hedges g − 0.54, 95% CI −0.90 to −0.18; *p* < 0.01), with no significant difference between modalities (*χ*² = 0.98, *p* = 0.32 for interaction). These effect sizes correspond approximately to reductions of ~1–2 mm in EAT thickness and ~5–15 mL in EAT volume across studies. SGLT2 inhibitor therapy was also associated with modest reductions in BMI (Hedges g − 0.23, 95% CI −0.43 to −0.03; *p* = 0.03) and systolic blood pressure (Hedges g − 0.32, 95% CI −0.59 to −0.05; *p* = 0.02).

### Sensitivity analyses (study design)

In analyses stratified by study design, a random-effects meta-analysis of five randomised controlled trials (*n* = 129) demonstrated that SGLT2 inhibitor therapy was associated with a significant reduction in EAT (Hedges g − 1.19, 95% CI −1.72 to −0.66; *p* < 0.001), with substantial heterogeneity across studies (*I*² = 79%). In contrast, observational studies (*n* = 155) showed a more modest but statistically significant reduction in EAT (Hedges g − 0.28, 95% CI −0.49 to −0.08; *p* = 0.007), with low between-study heterogeneity (*I*² = 18%). The smaller effect size observed in observational cohorts likely reflects greater variability in change-score measurements, heterogeneity in imaging protocols, and the inherent limitations of non-randomised study designs.

### Exploratory subgroup analysis (hypothesis-generating)

Exploratory subgroup analyses suggested a greater reduction in EAT in studies evaluating dapagliflozin compared with empagliflozin (Hedges g − 0.86 vs. −0.23; *p* = 0.002 for interaction). However, this represents an indirect comparison across a small number of heterogeneous studies (6 vs. 2 studies) with differing populations, imaging modalities, and follow-up durations, and should therefore be considered hypothesis-generating only, rather than evidence of drug superiority.

### Meta-regression analysis

Meta-regression analyses were performed to evaluate the influence of baseline covariates, including mean weight, body mass index, systolic blood pressure, LDL cholesterol, HbA1c, and eGFR. None of these variables were identified as significant moderators of treatment effect (*p* > 0.05 for all) (Table 4). Given the limited number of included studies, multivariable meta-regression incorporating multiple covariates was not performed due to the risk of overfitting.

**Table 4:** Meta regression analysis.

Moderator	Coefficient (95% CI)	*P*-value
Weight (kg)	0.01 (−0.02–0.03)	0.60
Body mass index	0.01 (−0.12–0.13)	0.94
Systolic blood pressure (mmHg)	−0.05 (−0.16–0.06)	0.26
LDL-cholesterol	0.04 (−0.02–0.09)	0.13
HbA_1_c	−0.37 (−0.79–0.04)	0.07
eGFR	0.02 (−0.06–0.09)	0.56

Meta-regression analysis of baseline moderators of EAT reduction.

Meta-regression analyses demonstrated that baseline clinical variables—including weight, body mass index, systolic blood pressure, LDL cholesterol, HbA1c, and eGFR—were not significant moderators of treatment effect (all *p* > 0.05). These findings should be interpreted with caution given the limited number of included studies.

### Qualitative endpoints

Several outcomes did not have sufficient data to permit quantitative synthesis and were instead summarised qualitatively.

#### Myocardial structure and function

One study demonstrated that empagliflozin reduced myocardial extracellular volume by approximately 1.25%, matrix volume by –7.24 mL (95% CI: –11.59 to –2.91), and cardiomyocyte volume by –11.08 mL (95% CI: –19.62 to –2.55) [22]. Another study using two-dimensional echocardiography reported an improvement in myocardial function as assessed by global longitudinal strain (−18.22 ± 2.35 at baseline vs. −19.13 ± 1.91 at 6 months) [31].

#### Systemic adiposity

Significant reductions were also observed in total body subcutaneous adipose tissue (SAT) area, with a mean change of –5.33 cm² (95% CI: –12.61–1.95) [22]. Similarly, another study found that visceral adipose tissue (VAT) decreased from 109 ± 44 to 97 ± 46 cm², and SAT decreased from 193 ± 71 to 177 ± 81 cm² after 3 months of SGLT2i therapy [23].

#### Inflammatory markers

SGLT2i therapy was also associated with reductions in inflammatory markers, including a decrease in TNF-α levels by 0.5 ± 0.7 pg/mL [26], and lower levels of C-reactive protein (CRP) [23]. Additionally, serum plasminogen activator inhibitor-1 (PAI-1) levels showed a non-significant trend toward reduction at 6 months, from 42.2 ± 16.1 to 32.9 ± 14.4 ng/mL (*p* = 0.07) [26].

## Discussion

The present meta-analysis consolidates evidence from eleven studies, demonstrating that SGLT2i significantly reduce both volumetric and linear measures of EAT, in addition to reductions in body mass index and systolic blood pressure. Exploratory analyses suggested larger reductions in EAT in studies evaluating dapagliflozin compared with empagliflozin; however, this indirect comparison should be interpreted as hypothesis-generating only, rather than evidence of drug superiority. These findings suggest the benefits extend beyond glucose lowering, highlighting a potential direct or pleiotropic cardiometabolic effect [32]. The observed decrease in EAT volume may also reflect associated reductions in systemic and local inflammation, as supported by concurrent improvements in inflammatory markers such as TNF-α, PAI-1 and CRP [33]. Obesity and visceral adiposity, both drivers of chronic low-grade inflammation, further perpetuate these effects, reinforcing the role of EAT as a mediator of cardiometabolic disease [34].

The present study provides several important incremental contributions beyond prior meta-analyses. First, we include contemporary evidence up to 2024–2025, incorporating newer randomised and mechanistic studies not included in earlier syntheses. Second, we provide a comprehensive cross-modality evaluation, integrating both volumetric (CT/CMR) and linear (echocardiographic) assessments of EAT, with modality-specific analyses to address measurement heterogeneity. Finally, we perform expanded subgroup and meta-regression analyses, enabling evaluation of potential modifiers such as drug class, baseline adiposity, and study design. Collectively, these additions provide a more contemporary, mechanistic, and methodologically robust synthesis compared with prior reports. Volumetric imaging using CT and CMR is more reproducible than echocardiographic thickness measurements [35], and therefore represents the primary imaging modality in this analysis, with echocardiographic measures presented as supportive data.

Therefore, these findings highlight the multifactorial benefits of SGLT2i and support the hypothesis that EAT serves as a marker and a modifiable mediator of cardiovascular risk. Reductions in EAT volume following SGLT2i therapy may explain the observed cardioprotective effects, providing a mechanistic link between metabolic modulation, inflammation, and clinical outcomes [36]. EAT has a unique anatomical position, surrounding the myocardium, which allows for direct paracrine signalling and release of inflammatory cascades [1]. EAT is known to play a critical role in the development of several cardiac diseases including coronary artery disease through atherosclerosis, heart failure through myocardial fibrosis and arrhythmogenesis [1]. As such, EAT is increasingly recognised not only as a biomarker of metabolic and cardiovascular risk but also as a potential therapeutic target [37].

SGLT2i represent a class of glucose-lowering agents with broad cardiorenal protective effects that transcend well beyond glycaemic control [38]. By inhibiting renal glucose reabsorption in the proximal tubules, SGLT2i induce glycosuria, leading to modest caloric loss and weight reduction [39]. Additionally, they promote natriuresis and osmotic diuresis, which reduces intravascular volume, lowers blood pressure, and decreases left ventricular preload and afterload [39]. These hemodynamic changes are important, as they are seen alongside reductions in left ventricular mass and improvements in diastolic function, thereby serving as the foundation for cardiovascular benefits. Beyond these effects, SGLT2i also enhance insulin sensitivity, lower serum uric acid, and improve markers of renal function and proteinuria [39]. Notably, in heart failure, the myocardium shifts toward using ketones, an oxygen-sparing substrate, which leads to improved energy efficiency and enhanced adenosine triphosphate availability [40]. These mechanisms support findings from major clinical trials including EMPA-REG OUTCOME [41], DAPA-HF [42], CREDENCE [43], CANVAS [44], and DECLARE-TIMI 58 [45] all which have demonstrated consistent reductions in heart failure hospitalisations and cardiovascular mortality.

Exploratory subgroup analyses suggested larger reductions in EAT in studies evaluating dapagliflozin compared with empagliflozin. While this observation raises the possibility of pharmacologic or mechanistic differences within the SGLT2 inhibitor class, potentially relating to differential effects on visceral adiposity, inflammatory pathways, or insulin sensitivity [46], this comparison is indirect and based on a small number of heterogeneous studies. Both agents are known to improve cardiometabolic parameters, and any apparent differences in EAT reduction should therefore be interpreted with caution. In the absence of head-to-head randomised data, these findings remain hypothesis-generating only and do not establish superiority of one agent over another.

The observed reductions in body mass index and systolic blood pressure with SGLT2i therapy were modest in the individual studies described, however may reflect physiological changes associated with loss of visceral and interstitial fat. Furthermore, the concurrent blood pressure lowering is also likely driven by natriuresis and improved arterial compliance.

Although a meta-analysis was not feasible for all cardiac parameters due to limited reporting of several indices, there are key insights emerge. Notably, empagliflozin was associated with a reduction in extracellular volume, matrix volume, and cardiomyocyte volume on magnetic resonance [22]. These changes suggest reverse myocardial remodelling, which is consistent with emerging evidence on SGLT2i promoting positive benefits in heart failure patients, independent of their glucose-lowering effects as shown in the major landmark trials.

Two studies demonstrated that SGLT2 inhibitor therapy was associated with reductions in both SAT and VAT. Notably, VAT is considered more metabolically and cardiometabolically deleterious than SAT due to its pro-inflammatory profile. At a molecular level, SGLT2 inhibitors appear to exert anti-inflammatory effects, with reductions in key biomarkers such as TNF-α, PAI-1, and C-reactive protein reported in two studies. These markers play central roles in inflammatory pathways implicated in atherosclerosis, adverse myocardial remodelling, and insulin resistance.

### Limitations

This meta-analysis has several limitations that should be considered when interpreting the findings. First, most studies had relatively short follow-up periods (≤6 months), which may not fully capture the long-term impact of SGLT2 inhibitors on adipose tissue remodelling. Second, whilst there was some heterogeneity identified in the pooled estimates, there were inherent differences in imaging techniques across studies to assess EAT volume, affecting spatial resolution, reproducibility and operator dependency. Third, the number of studies evaluating empagliflozin was limited to only two, which constrains the reliability of class-specific comparisons. In addition, class comparisons were based on indirect evidence, as no head-to-head trials between dapagliflozin and empagliflozin were available. Fourth, the meta-analysis included both prospective and retrospective observational studies, many of which had small sample sizes and short follow-up durations and therefore inherent biases such as selection and measurement bias cannot be fully excluded. Fifth, the limited number of included studies also precluded robust multivariable meta-regression, and therefore these analyses should be interpreted with caution due to the potential for overfitting and unstable estimates. Finally, none of the included studies reported intra- or inter-observer variability for EAT measurements.

## Conclusions

SGLT2 inhibitor therapy is associated with a reduction in epicardial adipose tissue, with consistent findings observed across randomised and observational data. These effects may contribute to the broader cardiometabolic benefits of this drug class; however, the clinical significance of EAT reduction remains to be established. Larger, longer-term studies are required to determine the durability of these changes and their relationship to cardiovascular outcomes.

## Supplementary information

Supplementary Materials
