# Supplementary material for: SGLT2 inhibitors are associated with reductions in epicardial adipose tissue volume and thickness: a meta-analysis
Source: Int J Obes (Lond). 2026 May 27;50(7):1398–407. doi: 10.1038/s41366-026-02110-6 (PMC13391349; doi:10.1038/s41366-026-02110-6)
Supplement: Supplementary file 1 — Supplementary Materials [file 41366_2026_2110_MOESM1_ESM.docx]

**SUPPLEMENTARY FILES**

**Supplementary File 1: Newcastle Ottawa Scale**

**
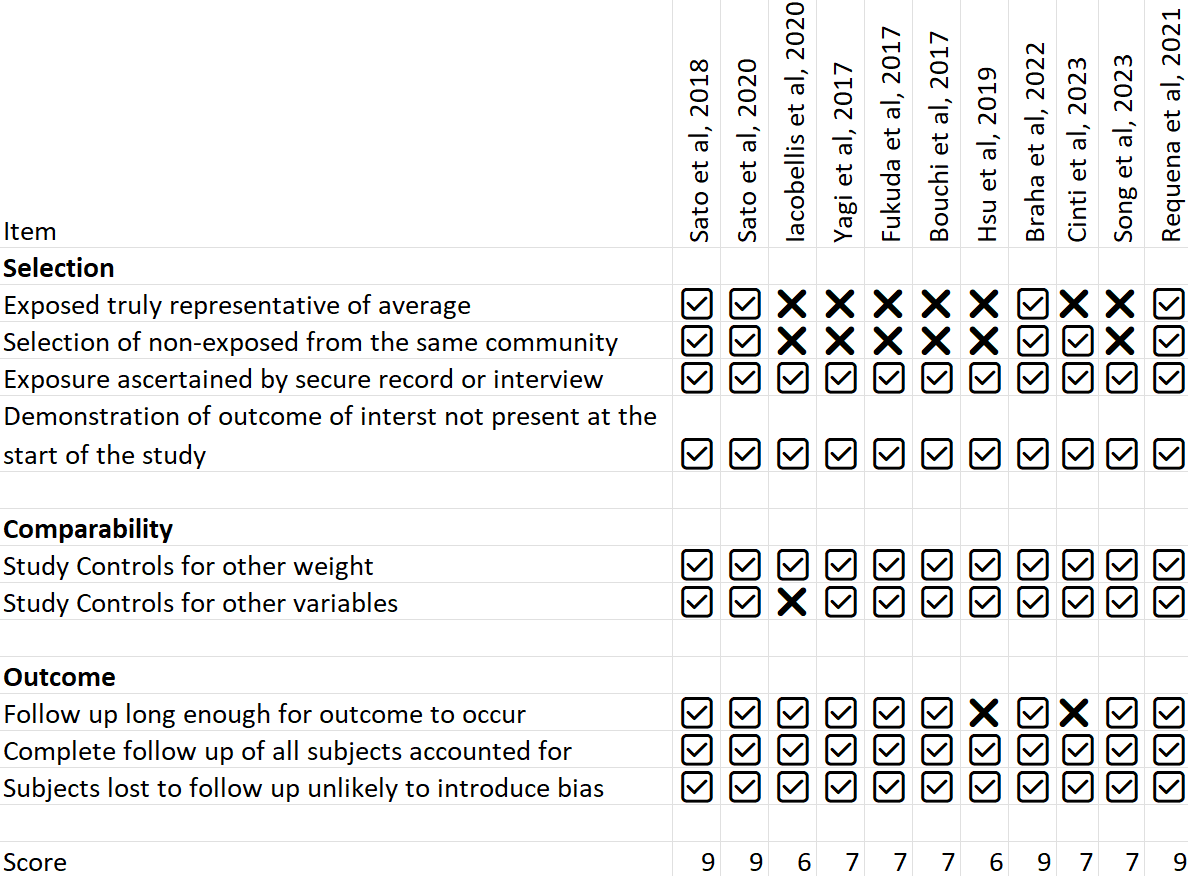
**

**Supplementary File 2: GRADE Evidence Profile**

| Study Design | No. of Studies | Participants (n) | Risk of Bias | Inconsistency | Indirectness | Imprecision | Publication Bias | Effect (Hedges g) | Certainty |
| --- | --- | --- | --- | --- | --- | --- | --- | --- | --- |
| Randomized Controlled Trials | 5 | 129 | Not serious | Serious¹ | Not serious | Not serious | Low | −1.19 (−1.72 to −0.66) | Moderate |
| Observational Studies | 6 | 155 | Serious² | Serious | Not serious | Serious³ | Low | −0.28 (−0.49 to −0.08) | Low |

Footnotes (GRADE justifications): ¹ Inconsistency (RCTs downgraded −1): There was substantial heterogeneity across trials (I² = 79%), likely reflecting differences in imaging modality (CT, CMR, echocardiography), study populations (T2DM vs HFrEF), and follow-up duration. ² Risk of Bias (observational studies downgraded −1): Most observational studies were single-arm or non-randomized, introducing risks of selection bias, confounding, and regression to the mean. ³ Imprecision (observational studies downgraded −1): Small sample sizes and wide variability in change-score standard deviations contributed to imprecision in effect estimates. Effect sizes correspond approximately to reductions of ~1–2 mm in EAT thickness and ~5–15 mL in EAT volume.

**Supplementary File 3A: Risk of bias assessment (Randomized controlled trials; RoB 2)**

| Study | Randomization process | Deviations from intended interventions | Missing outcome data | Measurement of outcome | Selection of reported result | Overall RoB 2 judgment |
| --- | --- | --- | --- | --- | --- | --- |
| Sato 2018 | Low | Some concerns | Low | Low | Some concerns | Some concerns |
| Sato 2020 | Low | Some concerns | Low | Low | Some concerns | Some concerns |
| Iacobellis 2020 | Low | Low | Some concerns | Low | Some concerns | Some concerns |
| Requena-Ibáñez 2021 | Low | Low | Low | Low | Low | Low risk |
| Cinti 2023 | Low | Low | Some concerns | Low | Some concerns | Some concerns |

**Supplementary File 3B: Risk of bias assessment (Non-randomized studies; ROBINS-I)**

| Study | Bias due to confounding | Bias in selection of participants | Bias in classification of interventions | Bias due to deviations from intended interventions | Bias due to missing data | Bias in measurement of outcomes | Bias in selection of reported result | Overall ROBINS-I judgment |
| --- | --- | --- | --- | --- | --- | --- | --- | --- |
| Yagi 2017 | Serious | Moderate | Low | Moderate | Low | Moderate | Moderate | Serious |
| Fukuda 2017 | Serious | Moderate | Low | Moderate | Low | Moderate | Moderate | Serious |
| Bouchi 2017 | Serious | Moderate | Low | Moderate | Low | Moderate | Moderate | Serious |
| Hsu 2019 | Serious | Moderate | Low | Moderate | Low | Moderate | Moderate | Serious |
| Braha 2022 | Serious | Moderate | Low | Moderate | Low | Moderate | Moderate | Serious |
| Song 2023 | Serious | Moderate | Low | Moderate | Moderate | Moderate | Moderate | Serious |

**Supplementary File 4: Comprehensive Search Strategy**

**Supplementary Comprehensive Search Strategy**

**1. PubMed (MEDLINE)**

(("Sodium-Glucose Transporter 2 Inhibitors"[Mesh] OR "SGLT2 inhibitor" OR "SGLT2 inhibitors" OR empagliflozin OR dapagliflozin OR canagliflozin) AND ("Epicardial Fat"[Mesh] OR "epicardial adipose tissue" OR "epicardial fat" OR "subepicardial fat" OR "subepicardial adipose tissue")) AND ("2000/01/01"[Date - Publication]: "2025/05/01"[Date - Publication])

**2. EMBASE (Ovid)**

('sodium glucose transporter 2 inhibitor'/exp OR 'sglt2 inhibitor':ti,ab OR empagliflozin:ti,ab OR dapagliflozin:ti,ab OR canagliflozin:ti,ab AND

('epicardial fat'/exp OR 'epicardial adipose tissue':ti,ab OR 'epicardial fat':ti,ab OR 'subepicardial fat':ti,ab OR 'subepicardial adipose tissue':ti,ab) AND [humans]/lim AND [2000-2025]/py

**3. Cochrane Library (CENTRAL)**

(("SGLT2 inhibitor" OR "sodium-glucose cotransporter 2 inhibitor" OR empagliflozin OR dapagliflozin OR canagliflozin OR ipragliflozin OR luseogliflozin) AND ("epicardial fat" OR "epicardial adipose tissue" OR "subepicardial fat" OR "subepicardial adipose tissue"))

**Supplementary File 5: FUNNEL PLOT (SGLT2i pre and post)**

**
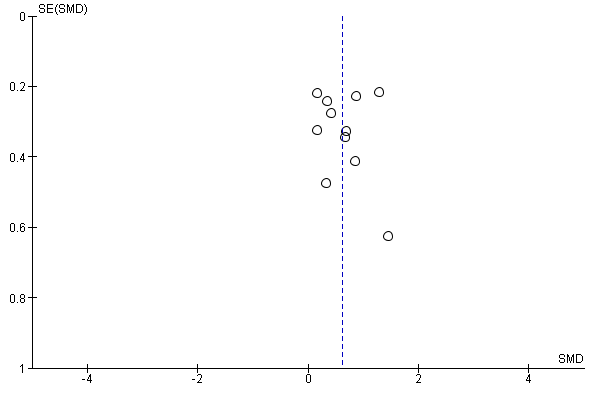
**

**Supplementary File 6: FUNNEL PLOT (SGLT2i vs controls)**

**
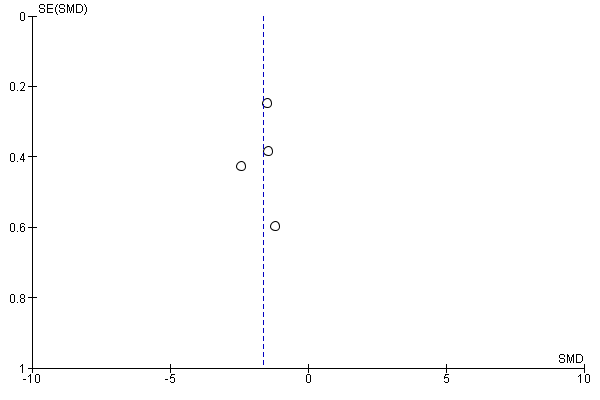
**
